# Minimal adverse outcomes of postnatal cytomegalovirus infection in term or moderate and late preterm infants

**DOI:** 10.3389/fped.2023.1048282

**Published:** 2023-02-03

**Authors:** Jie Chen, Yineng Zhou, Jie Tang, Chenyu Xu, Liping Chen, Biyun Xu, Yimin Dai, Yali Hu, Yi-Hua Zhou

**Affiliations:** ^1^ Department of Obstetrics and Gynecology, Nanjing Drum Tower Hospital, Nanjing University Medical School, Nanjing, China; ^2^ Department of Internal Medicine, Wuxi Children's Hospital, Wuxi, China; ^3^ Department of Obstetrics and Gynecology, Wujin Hospital Affiliated with Jiangsu University, Changzhou, China; ^4^ Department of Obstetrics and Gynecology, The Wujin Clinical College of Xuzhou Medical University, Changzhou, China; ^5^ Department of Obstetrics and Gynecology, Zhenjiang Fourth People's Hospital, Zhenjiang, China; ^6^ Department of Obstetrics and Gynecology, The First People's Hospital of Nantong, Nantong, China; ^7^ Medical Statistics and Analysis Center, Nanjing Drum Tower Hospital, Nanjing University Medical School, Nanjing, China; ^8^ Departments of Laboratory Medicine and Infectious Diseases, Nanjing Drum Tower Hospital, Nanjing University Medical School, Nanjing, China

**Keywords:** term or moderate or late preterm infants, cytomegalovirus, breast milk, delivery, clinical outcomes

## Abstract

**Objective:**

The aim of study was to investigate at what extent breastfeeding and vaginal delivery can increase mother-to-child transmission of cytomegalovirus (CMV) and to observe the clinical outcomes of postnatal infection in term or moderate and late preterm infants.

**Methods:**

In this retrospective study of prospectively collected clinical data and serum samples, during 2012–2015, 380 women with CMV IgG positive/IgM negative and their 384 infants (4 twin pairs) with gestational age ≥32 weeks were included. CMV IgG and IgM were measured with enzyme-linked immunosorbent assay.

**Results:**

Of 384 infants followed up at 10.2 ± 2.3 months age, 177 (46.1%) were defined with CMV infection based on the presence of higher CMV IgG levels than in their mothers. The infection rate in 190 breastfed infants was higher than in 194 formula-fed infants (62.6% vs. 29.9%, *P *< 0.001). Vaginally delivered infants (172) had higher CMV infection rate than 212 infants delivered by caesarean section (55.2% vs. 38.7%, *P *= 0.001). Compared with formula feeding and caesarean section, breastfeeding and vaginal delivery increased postnatal CMV infection respectively (OR = 3.801, 95% CI 2.474–5.840, *P *< 0.001; OR = 1.818, 95% CI 1.182–2.796, *P *= 0.007). Nevertheless, compared to uninfected infants, CMV-infected infants had comparable height and body weight and showed no adverse effect on the liver enzymes.

**Conclusion:**

Breastfeeding and vaginal delivery can increase postnatal CMV infection; however, the infection does not influence the growth of the term infants or preterm infants with gestational age ≥32 weeks. Thus, breastfeeding should be encouraged in these infants regardless of maternal CMV IgG status.

## Introduction

Human cytomegalovirus (CMV) infection is ubiquitous throughout the world. The prevalence of CMV IgG in women at childbearing age is 40%–70% in developed counties and >90%–100% in developing countries (1, 2). As more than 95% CMV IgG positive puerperants can shed virus in their breastmilk due to the viral reactivation in local breast glands (3), breastfeeding is considered to be the most common route for postnatal transmission from CMV-IgG positive mothers. This is in agreement with the fact that primary CMV infection mostly occurs in the first year of life (4, 5). However, it is unknown to what extent breastfeeding can increase the postnatal CMV infection in infants. Additionally, CMV may shed in vaginal secretion (6, 7), but the difference in postnatal CMV infection rates in infants delivered spontaneously or by cesarean section is less studied.

Generally, postnatal CMV infection in full term or late preterm infants does not have overt adverse outcomes, while postnatal CMV infection may cause illness in very early preterm (<32 gestation weeks) or very low birth weight (<1500 g) infants (8, 9). However, some reports show that postnatal CMV infection may also result in severe diseases in full term or late preterm infants (10–14). Thus, detection of CMV DNA in breast milk is sometimes performed in clinical practice in China, and mothers with positive CMV DNA in breastmilk are asked to avoid breastfeeding their infants (15).

In our previous prospective study on the mother-to-child transmission of hepatitis B, we collected paired serum samples from mothers and their infants, including cord blood samples from newborn infants, and recorded relevant maternal and infants' clinical data (16, 17). In the present study, we conducted a secondary analysis of the data from the paired mothers and infants by measuring CMV IgG and IgM to investigate at what extent that breastfeeding and vaginal delivery can influence the postnatal infection of CMV, and further observe the corresponding clinical outcomes of postnatal CMV infection in the infants.

## Materials and methods

### Subjects and Serum specimens

In our collaborative studies on the prevention of mother-to-child transmission of hepatitis B virus conducted in 5 hospitals in Jiangsu Province, China, between April 2012 and March 2015, we prospectively recruited 478 pregnant women with positive hepatitis B surface antigen (16, 17). Serum samples from these women during the second or third trimester of pregnancy were collected and kept at −30 °C. The data about the pregnancy, delivery, and neonatal outcomes were prospectively collected and recorded in a computerized database by the obstetricians at each participating hospital. The umbilical blood samples from newborn infants were also collected and kept at −30°C. All the neonates received recommended combined immunoprophylaxis against hepatitis B. In the follow-up study, maternal and infants' clinical data were recorded and serum samples from those women and their 418 infants (4 pairs of twins) were collected.

In the present study, we aimed to conduct a secondary analysis of data from the paired mothers and infants to investigate the influence of breastfeeding and vaginal delivery on postnatal infection of CMV, and further observe the infant's outcomes with postnatal CMV infection. Therefore, by further measuring CMV IgG and IgM of serum samples collected in the previous study (16, 17), we included 380 mothers who were CMV IgG positive/CMV IgM negative during the second or third trimester of pregnancy and their 384 (4 pairs of twins) infants in whom umbilical blood was CMV IgM negative in the present study. Those 19 women with both negative CMV IgG and IgM were not included. Fifteen paired mothers and infants were also excluded from analysis because there was insufficient serum volume. In the present study, perinatal information, including maternal gestational age, delivery mode, birth weight and height, and neonatal complications were extracted retrospectively from the originally computerized database collected during 2012–2015 (16, 17). Furthermore, in this study, we also retrieved the relevant data recorded in the previous follow-up study, including feeding mode, infant's age, height and weight, alanine aminotransferase (ALT) levels, and diseases requiring hospitalization such as pneumonia, severe gastroenteritis, or others. Formula-fed infants were fed exclusively with formula, while breastfed infants were defined as those who received exclusive breastfeeding or mixed feeding.

This study was approved by the ethics committee of Nanjing Drum Tower Hospital (No. 2012019) and all other participating hospitals. The study was performed in accordance with the ethical standards in the Declaration of Helsinki. All the pregnant women provided written informed consent and consented to follow up of their infants; each infant's informed consent was assigned by his/her mother in the previous study conducted in 2012–2015. Therefore, relevant data and serum samples of the mothers and their infants were used in the current study *via* an exemption approved by the institutional review board of each participating hospital.

### Quantification of CMV IgG and IgM

Serum samples were quantitatively tested for CMV IgG using a commercial enzyme-linked immunosorbent assay kit (Dia.Pro Diagnostic Bioprobes, Milano, Italy) as previously reported (4). The kit contains human plasma derived calibrators with CMV IgG at concentrations of 0, 0.5, 1, 2, 4, and 8 IU/ml. In the measurement, paired maternal and neonatal blood, diluted 1:101 with diluent (2% casein, 10 mM Tris-citrate buffer and 0.1% Tween 20), were tested in parallel, and positive and negative controls provided in the kit were also included. Calibration curve was established for each test, and the IgG level of serum samples was further quantified. Based on the manufacturer's instruction, the diluted sample with a concentration >0.5 IU/ml was considered positive for CMV IgG, thus, the sample with a concentration ≤0.5 IU/ml was considered negative. When the IgG concentration was beyond the upper detection limit (8 IU/ml), the sera were retested by further dilution.

CMV IgM was measured by the CMV IgM capture immunoassay (Dia.Pro Diagnostic Bioprobes). In each measurement, serum samples were diluted same as in detection of CMV IgG, and positive and negative controls were included as previously reported (4). As recommended by the manufacturer, the cut-off value was calculated as follows: cut-off = OD450 for negative control + 0.250. The test result was interpreted as a ratio of the sample OD450 and the cut-off value (S/Co). The sample was considered positive if S/Co value was >1.2, indeterminate if it was 1.0–1.2, and negative if it was <1.0. The indeterminate sample was retested; the sample was considered positive if S/Co value was 1.0–1.2 or >1.2, and negative if it was <1.0.

### Criteria for postnatal CMV infection in infants

Generally, diagnosis of postnatal CMV infection in infants is established based on the detection of CMV DNA by PCR in urine or saliva samples, but not based on the detection of CMV IgG, because infants have maternally derived IgG antibodies due to the transplacental transfer of maternal IgG. In the present study, we were unable to diagnose postnatal CMV infection with PCR because urine or saliva samples were unavailable. Previously, we showed that maternal CMV IgG in infants without postnatal infection disappeared at the age of 7 months (4). Moreover, since the half-life of IgG antibody is 21–24 days, if infants do not have postnatal CMV infection, they should have much lower CMV IgG levels than their mothers after the age of 7 months. Therefore, in the present study, we determined postnatal CMV infection in infants at the age of 7 months or older when they had higher CMV IgG levels than their mothers.

### Statistical analysis

Data were analyzed with the SPSS software (SPSS Standard version 11.0, SPSS Inc., Chicago, IL, USA). Continuous variables normally distributed were expressed as mean ± standard deviation and compared by two-sample or paired *t*-test. Quantitative data non-normally distributed are presented as median and range, and compared by Man–Whitney *U* test. Categorical variables were reported as number and percentage, and compared by *χ*^2^ analysis or Fisher's exact test where appropriate. Logistic regression analyses were further performed to determine the independent role of the feeding and delivery mode in postnatal CMV infection of the infants; the results were expressed by the adjusted odds ratios (OR) with 95% confidence intervals (CI). A two-sided *P* value fewer than 0.05 was considered statistically significant.

## Results

### General characteristics of the study subjects

In total, 380 mothers with CMV IgG positive/IgM negative during the second or third trimester of pregnancy and their 384 infants (4 twin pairs) who were CMV IgM negative in umbilical blood were included in the study. The women were 27.5 ± 4.3 years old at delivery, and their infants were at the age of 10.2 ± 2.3 months at follow-up. Of these 384 infants, 190 (49.5%) were breastfed and 194 (50.5%) others were exclusively formula-fed. The mothers of breastfed and formula-fed infants had comparable ages (27.9 ± 4.1 vs. 27.1 ± 4.4, *P *= 0.068) and had comparable gestational ages at delivery (38.9 ± 1.4 vs. 38.7 ± 1.7, *P *= 0.213). The breastfed and formula-fed infants had comparable boy/girl ratios (formula-fed 109/83 vs. breastfed 104/82) and ages (formula-fed 10.5 ± 2.9 vs. breastfed 9.8 ± 2.2 months), although the difference of age had statistical significance (*P *= 0.008).

Of the 384 infants, 362 (94.3%) were term infants with the gestational age ≥37 weeks and 22 (5.7%) others were moderate and late preterm infants with the gestational age 32 to 36 + 6 weeks. Of the preterm infants, 14 received exclusive formula feeding and 8 received breastfeeding.

### Seroprevalence of CMV IgG in infants with different feeding patterns

Previous study showed that transplacentally transferred maternal CMV IgG in infants disappears before 6–8 months age because of the natural IgG decay (4, 18). In the present study, all infants participated were over 7 months age, therefore, we measured the CMV IgG as a marker of CMV infection in the infants. Of the 384 infants at follow-up, 177 (46.1%) were CMV IgG positive and 207 others (53.9%) were negative (Table 1). As shown in Table 1, 62.6% (119/190) of the breastfed infants were CMV IgG positive, much higher than the positive rate of 29.9% (58/194) in the formula-fed infants (*χ*^2 ^= 41.403, *P *< 0.001). Overall, the seroprevalence of CMV IgG in breastfed group at different ages was each higher than that in formula-fed infants (Table 1). However, in either of breastfed or formula-fed groups, the seroprevalence of CMV IgG was relatively constant in infants at the age of different months, and did not show an increasing trend with growing ages (Table 1).

**Table 1:** Seroprevalence of cytomegalovirus (CMV) IgG in breast- and formula-fed infants.

Age (months)	Overall		Breastfed^a^		Formula-fed^a^		*P*
	*n*	IgG + (%)	*n*	IgG + (%)	*n*	IgG + (%)	
7–8	92	46 (50.0)	47	31 (66.0)	45	15 (33.3)	0.002
9–10	172	80 (46.5)	91	59 (64.8)	81	21 (25.9)	<0.001
11–12	85	35 (41.2)	39	22 (56.4)	46	13 (28.3)	0.009
13–26	35	16 (45.7)	13	7 (53.8)	22	9 (40.9)	0.458
Total	384	177 (46.1)	190	119 (62.6)	194	58 (29.9)	<0.001

^a^ The prevalence of CMV IgG among infants at different age had no statistical difference either in breastfed infants (*χ*^2 ^= 1.62, *P *= 0.654) or in formula-fed infants (*χ*^2 ^= 2.19, *P *= 0.533).

To further validate the CMV IgG in infants was resultant from the infection, rather than from the transplacentally acquired maternal IgG, we compared the CMV IgG levels between each CMV IgG positive child and his/her mother. Each CMV IgG positive child had a higher level than his/her mother. The mean concentrations in infants at different age subgroups were higher than those in their mothers, especially in 7–8 and 9–10 months age groups (Table 2), demonstrating that the CMV IgG in these infants were acquired by postnatal CMV infection. Additionally, we tested CMV IgM in the 177 CMV IgG positive infants, and 36 (20.3%) infants were positive, with 25 (21.0%) from the 119 breastfed group and 11 (19.0%) from the 58 formula-fed group (Table 3).

**Table 2:** Comparison of cytomegalovirus IgG levels (IU/ml) between the infants and mothers.

Age (months)	Infant		Mother		*P*
	*n*	Concentration	*n*	Concentration	
7–8	46	559.8 ± 239.0	45	400.0 ± 256.3	0.003
9–10	80	549.2 ± 262.1	77	426.7 ± 249.7	0.003
11–12	35	492.6 ± 255.2	35	427.2 ± 207.0	0.243
13–26	16	508.2 ± 204.6	16	386.6 ± 242.4	0.136
Total	177	537.1 ± 249.5	173^a^	416.2 ± 241.2	<0.001

^a^ Four mothers delivered twin infants.

**Table 3:** Seroprevalence of cytomegalovirus (CMV) IgM in breast- and formula-fed infants with positivity of CMV IgG.

Age (months)	Overall		Breastfed		Formula-fed		*P* ^a^
	*n*	IgM + (%)	*n*	IgM + (%)	*n*	IgM + (%)	
7–8	46	12 (26.1)	31	10 (32.3)	15	2 (13.3)	0.312
9–10	80	15 (18.8)	59	11 (18.6)	21	4 (19.0)	>0.999
11–12	35	6 (17.1)	22	3 (13.6)	13	3 (23.1)	0.801
13–26	16	3 (18.8)	7	1 (14.3)	9	2 (22.2)	>0.999
Total	177	36 (20.3)	119	25 (21.0)	58	11 (19.0)	0.751

^a^ Comparison between breastfed and formula-fed infants.

### Seroprevalence of CMV IgG in infants with different delivery modes

CMV may exist in genital secretions of CMV IgG positive women (6, 7). To clarify whether vaginal delivery can increase the likelihood of mother-to-child transmission of CMV, we compared the positivity rate of CMV IgG in 172 vaginally delivered infants with that in 212 infants delivered by cesarean section (Table 4). The overall positive rate of CMV IgG in vaginally delivered infants was higher than that in those delivered by caesarean section (55.2% vs. 38.7%, *χ*^2 ^= 10.474, *P *= 0.001). Further stratified analysis showed that while the breastfed infants had similar positive rate of CMV IgG between virginally delivered infants and those delivered by cesarean section (65.3% vs. 60.0%, *P *= 0.453), formula-fed infants delivered virginally had a significantly higher positive rate of CMV IgG than formula-fed infants delivered by caesarean section (42.9% vs. 21.4%, *P *= 0.001). Further logistic regression analysis showed that, compared with formula feeding, breast milk could increase the seropositive rate of CMV in the infants (OR = 3.801, 95% CI 2.474–5.840, *P *< 0.001). Similarly, vaginal delivery was also independently associated with higher seroprevalence of CMV in the infants (OR = 1.818, 95% CI 1.182–2.796, *P *= 0.007).

**Table 4:** Seroprevalence of cytomegalovirus (CMV) IgG in infants who were born by vaginal delivery or caesarean section.

	Vaginal delivery		Caesarean section		*P*
	*n*	CMV IgG + (%)	*n*	CMV IgG + (%)	
Breastfed	95	62 (65.3)	95	57 (60.0)	0.453
Formula-fed	77	33 (42.9)	117	25 (21.4)	0.001
Total	172	95 (55.2)	212	82 (38.7)	0.001

### Influence of postnatally acquired CMV infection on infants' height and body weight

To determine whether postnatally acquired CMV infection may affect the infants' growth, we compared the infants' weight and height between the CMV IgG-positive and -negative infants respectively. The infants between the two groups had comparable birth weights and heights, gender ratios and ages. Table 5 shows that the CMV IgG-positive and -negative infants had comparable body weights and heights at follow-up. Of the 362 term infants and 22 moderate/late preterm infants, 171 (47.2%) and 6 (27.3%) were defined with postnatal infection at follow-up respectively (*P* = 0.068), indicating that moderate/late preterm infants did not have an increased risk for postnatal CMV infection. Further comparison between the term and moderate/late preterm infants showed that the infected preterm infants had similar mean height and body weight at follow-up (Table 5).

**Table 5:** Infants’ parameters with respect to the status of cytomegalovirus (CMV) IgG.

	Total			Term			Preterm		
	CMV IgG + (*n* = 177)	CMV IgG − (*n* = 207)	*P*	CMV IgG + (*n* = 171)	CMV IgG − (*n* = 191)	*P*	CMV IgG + (*n* = 6)	CMV IgG − (*n* = 16)	*P*
Age (months)	10.1 ± 2.9	10.2 ± 2.3	0.707	10.1 ± 2.9	10.3 ± 2.6	0.489	9.7 ± 1.5	10.1 ± 1.8	0.634
Male (%)	102 (57.6)	111 (53.6)	0.431	100 (58.5)	102 (53.4)	0.332	2 (33.3)	9 (56.3)	0.632
Height (cm)	75.67 ± 4.97	74.94 ± 5.16	0.161	75.68 ± 4.93	74.92 ± 5.22	0.157	74.76 ± 4.64	75.14 ± 4.88	0.871
Weight (kg)	10.86 ± 2.23	10.54 ± 2.12	0.151	10.87 ± 2.22	10.55 ± 2.12	0.162	10.12 ± 1.98	10.34 ± 1.92	0.815

CMV IgG positive alone usually represents past CMV infection and positivity for both CMV IgG and IgM usually indicates an active infection of CMV. At follow-up, a total of 36 infants were found to be both CMV IgG and IgM positive, however, these infants did not have particular clinical manifestation. Of them, 33 had measured ALT level and 3 others did not measure ALT due to the hemolysis; 5 (15.1%) infants had mild elevation of ALT (42.0–107.2 U/L) without overt clinical presentations. Of the 98 infants with positive CMV IgG alone and 144 infants with negative CMV IgG, 14 (14.3%) and 20 (13.9%) also had mild elevation of ALT respectively. The ALT abnormal rates among these three groups of infants had no statistical difference (*χ*^2 ^= 0.036, *P *= 0.982). In addition, infants with postnatal CMV infection had no records of severe diseases requiring hospitalization, compared with the infants without postnatal CMV infection.

## Discussion

In the present study, we showed that 46.1% (177/384) of infants born to mothers with positive CMV IgG and negative CMV IgM experienced postnatal CMV infection at the age of 10.2 ± 2.3 months, and breastfeeding and vaginal delivery each increased the mother-to-child transmission of CMV, with the OR 3.801 and 1.818 respectively. However, CMV-infected and uninfected infants had comparable body weights and heights at follow-up, and CMV-infected infants did not experience more diseases requiring hospitalization. This study indicates that breastfeeding should not be contraindicated for infants born to CMV IgG positive parturients, even as high as 96% of CMV IgG positive mothers have CMV in breast milk (3).

In this study, we defined the postnatal CMV infection based on the higher CMV IgG levels in infants than in their mothers. One may assume that the CMV IgG in infants is derived from the mothers because of the transplacental transfer of maternal CMV IgG. However, our previous study and others demonstrated that the maternally acquired CMV IgG in infants disappears before the age of 6–8 months (4, 18). In the present study, all infants were negative for CMV IgM at birth and were positive for CMV IgG over the age of 7 months. Moreover, the CMV IgG level in each infant was higher than that in the mother, indicating that the presence of CMV IgG in the infant was not derived from mothers, but was resulted from the postnatal CMV infection. Although CMV IgM negativity in infants at birth does not necessarily exclude congenital infection, the overall congenital CMV in infants in China is lower than 0.5% (19, 20). Therefore, the presence of relatively higher levels of CMV IgG can define the postnatal CMV infection in vast majority of these infants, if not all.

Of the all 177 CMV IgG positive infants, 36 (20.3%) showed CMV IgM positive (Table 3). The positive rate of CMV IgM appears to be lower, since the mean age of these CMV IgG positive infants was only 10.1 ± 2.9 months and the CMV infection in these infants should be primary. However, our previous study with detection of CMV IgG and IgM in longitudinal serum samples at ages of 1, 3.5, 8, and 24 months in a cohort of infants showed that CMV IgM was mostly positive (83.3%) at ages of 1 and 3.5 months (4), indicating the postnatal CMV infection mostly occurs before 3.5 months age. Considering that CMV IgM usually disappears within 1–3 months, ∼20% of CMV IgM positive infants in the present study were logically reasonable.

The critical findings of our present study are that, compared with the infants who did not experience postnatal CMV infection, the CMV infected infants had similar body weights and heights (Table 5) and did not have more severe diseases requiring hospitalization, and that the infants with active primary CMV infection (positive for both CMV IgG and IgM) had a similar rate of ALT elevation, compared to the infants with latent CMV infection (CMV IgG positive alone) and the infants without CMV infection (negative for both CMV IgG and IgM). These results indicated that postnatal infection of CMV in infants with gestational age ≥32 weeks does not cause obvious adverse influence. The minimal adverse influence may be associated with the presence of maternal CMV IgG in infants, which can neutralize the virulence of CMV and provide substantial protection against symptomatic diseases or sequelae (21).

The results of our study have several practical implications. First, for newborn infants with gestational age ≥32 weeks, the presence of CMV DNA in breast milk should not be the contraindication for breastfeeding (22). Thus, detection of CMV DNA in breast milk of CMV IgG-positive puerperants who delivered her neonates after 32 weeks gestation is excessive and is not necessary. Second, it should be cautious to use saliva as detection materials in defining *in utero* infection of CMV. The optimal approach to diagnosing congenital CMV infection is to detect CMV DNA in urine of newborns within 3 weeks after birth (23, 24). However, due to the difficulty in collecting neonatal urine samples, saliva samples are usually used to detect CMV DNA (25–27). In the present study, we found that the infants who were delivered vaginally and/or breastfed had higher CMV infection rate, indicating that neonatal saliva samples may be contaminated with maternal CMV. Thus, CMV DNA in saliva samples from newborns who were delivered virginally and/or breastfed may be false positive caused by contamination of maternal CMV (27–29). Indeed, the congenital infection rate estimated by detection of CMV DNA in saliva appears to be higher than that based on detecting CMV DNA in urine (24, 30). Third, the findings that 29.9% of the exclusively formula-fed infants were infected with CMV (Table 1) and the higher CMV infection in vaginally delivered infants (Table 4) indicate that the CMV infection in infants is not necessarily caused by breastfeeding. Thus, breastfeeding should not be given up to avoid CMV infection, particularly in very early preterm (<32 gestation weeks) or very low birth weight (<1500 g) infants.

There are several limitations in this study. First, we excluded congenital CMV infection just by detecting CMV IgM in cord blood, but not by detecting CMV DNA in urine samples of the newborns within three weeks after birth (31). Second, the postnatal CMV infection routes other than breastfeeding and vaginal delivery should also be considered, such as horizontal infection in daycare center. However, in our country, young infants are mostly taken care by mothers or grandparents at home, but not in the kindergarten. Third, because of the retrospective analysis of the data, we were not able to longitudinally investigate the clinical features, such as liver function and platelet counts, in the infants during acute infection phase. Therefore, we could not confirm whether acute CMV infection in those infants has caused relevant symptomatic diseases. Fourth, we did not follow up detailed neurological parameters of infants, thus the long term effect of postnatal CMV on infants' neurological development requires further observation.

In conclusion, the results of this study showed that breastfeeding and vaginal delivery can increase the risk of postnatal CMV infection; however, the infection does not cause obviously adverse events in the infants born over 32 gestational weeks. Therefore, detection of CMV DNA in breastmilk should not be routinely performed and breast-feeding should be recommended.

## Data Availability

The raw data supporting the conclusions of this article will be made available by the authors, without undue reservation.
